# FRACTION: protocol of a phase II study of Fedratinib and Nivolumab combination in patients with myelofibrosis and resistance or suboptimal response to JAK-inhibitor treatment of the German MPN study group (GSG-MPN)

**DOI:** 10.1007/s00277-024-05867-w

**Published:** 2024-07-05

**Authors:** Susanne Isfort, Nikolas von Bubnoff, Haifa Kathrin Al-Ali, Heiko Becker, Thorsten Götze, Philipp le Coutre, Martin Griesshammer, Claudia Moskwa, Luisa Wohn, Johanna Riedel, Francesca Palandri, Kirsi Manz, Andreas Hochhaus, Konstanze Döhner, Florian H. Heidel

**Affiliations:** 1 Hematology, Hemostasis, Oncology and Stem Cell Transplantation, Hannover Medical School (MHH), Carl-Neuberg-Str. 1, Hannover, 30625 Germany; 2 Department of Hematology and Oncology, University Hospital Lübeck, Lübeck, Germany; 3 University Hospital Halle (Saale), Krukenberg Cancer Center Halle, Halle, Germany; 4 Department of Medicine I - Medical Center - University of Freiburg, Faculty of Medicine, University of , Freiburg, Freiburg, Germany; 5 Krankenhaus Nordwest, University Cancer Center (UCT), Frankfurt, Germany; 6 Institut für Klinische Krebsforschung IKF Am Krankenhaus Nordwest, Frankfurt, Germany; 7 Department of Hematology, Oncology and Stem Cell Transplantation, Charite Berlin, Germany; 8 Mühlenkreisklinikum Minden, Universitätsklinikum Bochum, Minden, Germany; 9 Internal Medicine C, Hematology, Oncology, Stem Cell Transplantation and Palliative Care, University Medicine Greifswald, Greifswald, Germany; 10 IRCCS Azienda Ospedaliero-Universitaria Di Bologna, Istituto Di Ematologia “Seràgnoli”, Bologna, Italy; 11 Institute for Community Medicine – SHIP-KEF, Universitätsmedizin Greifswald, Greifswald, Germany; 12 Klinik für Innere Medizin II, Abteilung Hämatologie und Internistische Onkologie, Universitätsklinikum Jena, Jena, Germany; 13 Department of Internal Medicine III, University Hospital of Ulm, Ulm, Germany

**Keywords:** Myeloproliferative Neoplasms (MPN), Myelofibrosis (MF), Checkpoint-inhibitor, Fedratinib, Nivolumab, Disease modification

## Abstract

Development of Janus-kinase (JAK) inhibitors has revolutionized the therapeutic landscape for patients with myeloproliferative neoplasia (MPN). Following approval of the first JAK1/2-inhibitor Ruxolitinib, symptoms of this inflammatory disease, characterized by splenomegaly, release of inflammatory cytokines and appearance of thrombosis, could be effectively reduced for the first time. However, JAK-inhibitor treatment is limited in several aspects: 1) duration of response: 3 years after initiation of therapy more than 50% of patients have discontinued JAK-inhibitor treatment due to lack of efficacy or resistance; 2) reduction of disease burden: while effective in reducing inflammation and constitutional symptoms, JAK-inhibitors fail to reduce the malignant clone in the majority of patients and therefore lack long-term efficacy. Early clinical trials for patients with myelofibrosis (MF) have tried to address these issues for patients with suboptimal response to Ruxolitinib therapy while combination therapies with Fedratinib are rare. Recent reports provided first evidence on how the JAK2-V617F mutated myeloid cells may influence T-cell responses. JAK2-V617F promoted the synthesis of PD-L1 in MPN cells leading to limited anti-neoplastic T-cell responses, metabolic changes in T-cells and eventually JAK2-V617F-driven immune-escape of MPN cells. These findings may facilitate the use of immunotherapeutic approaches for JAK-mutated clones. Immune checkpoints refer to a variety of inhibitory pathways that are crucial for maintaining self-tolerance and modulating the duration and amplitude of physiological immune responses in peripheral tissues in order to minimize collateral tissue damage. The FRACTION study is a single arm, open label Phase II trial investigating the combination of Fedratinib with the PD-1 inhibitor Nivolumab in patients with myelofibrosis and suboptimal or lack of response to JAK-inhibitor therapy. Over a 12 months period the trial assesses longer term outcomes, particularly the effects on clinical outcomes, such as induction of clinical remissions, quality of life and improvement of anemia. No prospective clinical trial data exist for combinations of JAK- and immune-checkpoint-inhibitors in the planned MF study population and this study will provide new findings that may contribute to advancing the treatment landscape for MF patients with suboptimal responses and limited alternatives.

**Supplementary Information:**

The online version contains supplementary material available at 10.1007/s00277-024-05867-w.

## Trial registration

EudraCT Number 2021-004757-23.

**Supplementary Information:**

The online version contains supplementary material available at 10.1007/s00277-024-05867-w.

## Introduction

Development of Janus-kinase inhibitors has revolutionized the therapeutic landscape for patients with myeloproliferative neoplasia (MPN). Following approval of the first Jak1/2-inhibitor Ruxolitinib (Rux), symptoms of this inflammatory disease, characterized by splenomegaly, release of inflammatory cytokines and appearance of thrombosis, could be effectively reduced for the first time [1]. However, JAK-inhibitor treatment is limited in several aspects: (i) duration of response: 3 years after treatment more than 50% of patients have discontinued JAK-inhibitor treatment due to lack of efficacy or resistance [2, 3]. (ii) reduction of disease burden: while effective in reducing inflammation and constitutional symptoms, JAK-inhibitors fail to reduce the malignant clone in the majority of patients and therefore lacks long-term efficacy [4].

Clinical trials for patients with myelofibrosis (MF) have tried to address these issues for patients with suboptimal response to Ruxolitinib therapy. Combination therapies have not been evaluated with Fedratinib so far. Potential combination partners include signaling inhibitors, epigenetic drugs and immunotherapeutics, among others.

The consequences of JAK-inhibition on human immune cell function have been studied in more detail. In early clinical trials, increased numbers of viral infections have been described in MPN patients on treatment with the JAK1/2 inhibitor Ruxolitinib [5, 6]. Moreover, JAK1/2-inhibitors such as Ruxolitinib have been used successfully in rheumatological diseases as well as in the setting of allogeneic transplantation as treatment of acute and chronic graft-versus-host disease (GvHD). In murine models of GvHD Ruxolitinib enhanced survival and limited proinflammatory cytokine production as well as Th1 and Th17 polarization. In MPN patients, JAK1/2 inhibition leads to reduction in CD3 + T-cells and decreased cytokine production. Within the T-cell compartment regulatory T-cells (Treg) and Th1 cells seem to be most prominently affected [7–9]. Also, effector functions of CD8 + T-cells are impaired upon JAK1/2 inhibitor treatment [10]. Furthermore, JAK-inhibition compromises B-cell differentiation and antibody production [11, 12] as well as dendritic cell function [13]. Therefore, it is crucial to determine whether inhibitors targeting JAK1, JAK2, or both could impair T-cell function. JAK1/2 inhibitors as well as JAK2 specific inhibitors had been studied in vitro and in vivo on healthy donor T-cells. Exposure to either JAK1/2 inhibitor resulted in the inhibition of proliferation, global activation (CD69), and STAT1 phosphorylation of CD4 + and CD8 + T-cells, while selective JAK2 inhibition had no such effect. Likewise, when using genetic inactivation of either JAK1 or JAK2 in T-cells only JAK1 depletion was sufficient to inhibit global T-cell function in vitro. JAK2 was dispensable for global T-cell effector functions in mouse models of GvHD. These findings underscore the importance of JAK-selectivity depending on the underlying condition or context. [14]. Consistently, while there were signals for increased rates of infectious complications upon Ruxolitinib treatment in clinical trials, there was no increased incidence in trials using Fedratinib [6, 15–17], with a relatively low rate of infectious complications. These findings indicate that JAK2-specific inhibitors like Fedratinib allows immune responses and therefore may serve as a combination partner for immunotherapeutic agents.

Fedratinib is an oral wild type and mutated Janus Kinase 2 (JAK2) and (FLT3) inhibitor. Fedratinib is JAK2-selective with higher potency for JAK2 over family members JAK1, JAK3 and TYK2. In cell models expressing mutationally active JAK2 or FLT3, Fedratinib reduced phosphorylation of STAT3 and 5 proteins, inhibited cell proliferation, and induced apoptosis. In mouse models of JAK2V617F-driven myeloproliferative neoplasms, Fedratinib inhibited phosphorylation of STAT3/5, increased survival and improved MPN-associated symptoms. This included reduction of white blood cells, hematocrit, splenomegaly, and fibrosis. Fedratinib has been studied extensively in the treatment of patients with myelofibrosis and has recently demonstrated clinical efficacy in a randomized, placebo-controlled, Phase 3 study (JAKARTA) in patients with intermediate-2 or high-risk MF [18, 19].

Most notably, recent reports provided first evidence on how the JAK2-V617F mutated myeloid cells may influence T-cell responses. JAK2-V617F promoted the synthesis of PD-L1 in MPN cells leading to limited anti-neoplastic T-cell responses, metabolic changes in T-cells and eventually JAK2-V617F-driven immune-escape of MPN cells [20]. These findings may facilitate the use of immunotherapeutic approaches for JAK-mutated clones. Immune checkpoints refer to a variety of inhibitory pathways that are crucial for maintaining self-tolerance and modulating the duration and amplitude of physiological immune responses in peripheral tissues in order to minimize collateral tissue damage. Checkpoint inhibitors, such as ipilimumab targeting CTLA-4, pembrolizumab and Nivolumab targeting programmed death-1 (PD-1), have been approved for numerous cancer indications including hematologic malignancies. Nivolumab is one of the most extensively studied immune checkpoint inhibitors across various tumor types. Nivolumab was the first PD-1 immune checkpoint inhibitor to be approved for use in advanced, squamous non-small cell lung cancer (NSCLC) following prior chemotherapy. Nivolumab is a human immunoglobulin G4 (IgG4) monoclonal antibody. It binds to the PD-1 receptor and blocks its interaction with PD-L1 and PD-L2, thus releasing PD-1 pathway-mediated inhibition of the immune response, including the antitumor immune response.

Checkpoint inhibitors Nivolumab and Pembrolizumab have been investigated as monotherapy in patients with myelofibrosis. However, treatment duration was rather short due to the lack of early responses. Of note, immune responses upon checkpoint inhibitor treatment may require a prolonged treatment period to facilitate measurable clinical responses. Upon monotherapy, changes in the immune milieu could still be recorded, further suggesting that a prolonged treatment may facilitate improved responses. Therefore, Fedratinib was a logical combination partner for checkpoint inhibitors as it is an effective JAK-inhibitor to reduce spleen size and symptoms while allowing immune responses over a prolonged period of treatment.

The aim of this phase 2 trial is therefore to evaluate the clinical efficacy of Fedratinib and Nivolumab combination therapy in patients with primary and secondary MF based on the consensus criteria of the International Working Group for Myelofibrosis Research and Treatment (IWG-MRT), extended by the criterion RBC-transfusion independence (RBC-TI).

## Methods

### Study design and objectives

**Fig. 1:**
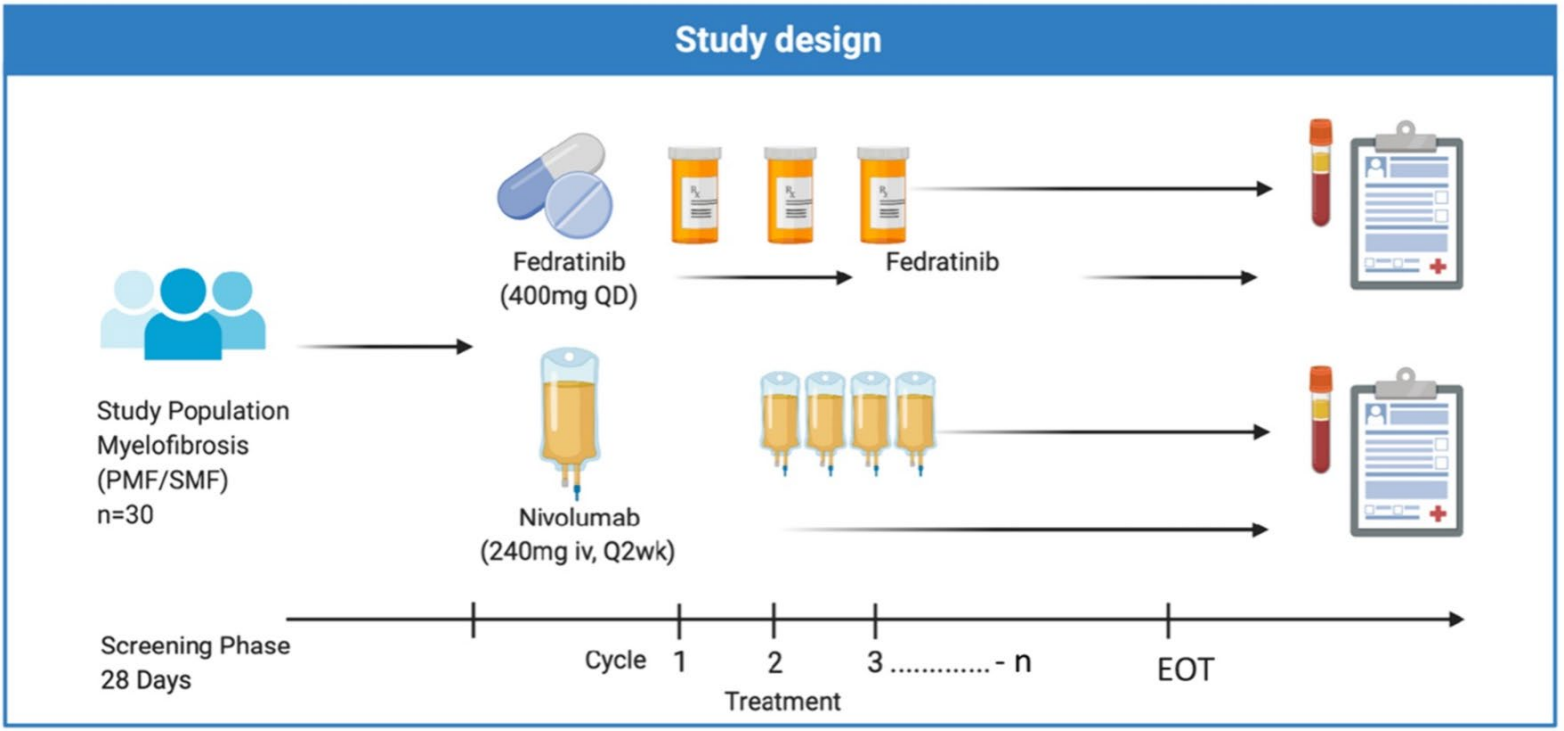
Study design. Cycle 1 consists of a run-in phase of Fedratinib monotherapy (400 mg QD) followed by addition of Nivolumab (240 mg Q2wk) from cycle 2 on. Patients will receive study treatment until progressive disease or relapse (acc. to IWG-MRT), death, or study discontinuation for other reasons. Treatment discontinuation will be required upon progression, toxicity or due to patient decision

This study is an open-label, single-arm, phase-II trial to assess the efficacy of a drug combination of Fedratinib and Nivolumab in primary (pre-fibrotic or overt) and secondary MF patients with no response or suboptimal response to any JAK-inhibitor therapy (regarding persistence of symptoms, splenomegaly, cytopenia or hyperproliferation) OR failure [secondary resistance] to JAK-inhibitor treatment as defined by IWG-MRT criteria. Dosing regimen of the drugs is derived from previous studies with either Fedratinib or Nivolumab. Fedratinib treatment will be started at 400 mg QD, whereas the dose of Nivolumab will be started in cycle 2 at a fixed dose of 240 mg i.v. every 2 weeks (Fig. 1). Dose adjustment or discontinuation is intended for both drugs in case of severe hematologic and/or non-hematologic toxicity. Treatment cycles are defined as 28 days. The estimated total duration of the study for each patient is 12 months. Patients whose disease responds or who show at least a clinical benefit may continue to receive the combination. Patients will receive study treatment until progressive disease or relapse (acc. to IWG-MRT), death or study discontinuation for other reasons. Both drugs do not have an overlapping toxicity profile as they target distinct biological pathways (JAK2-inhibitor; immune-checkpoint inhibitor). Both classes of compounds have been investigated in phase 2 and 3 trials without excess of toxicity [21–27].

Primary objective is the evaluation of the clinical efficacy of Fedratinib and Nivolumab combination therapy in primary and secondary MF patients based on the consensus criteria of the International Working Group for Myelofibrosis Research and Treatment (IWG-MRT) [28], extended by the criterion RBC-transfusion independence (RBC-TI) [29, 30]. Secondary objectives include evaluation of the safety of a combination therapy (Fedratinib and Nivolumab) in patients with primary and secondary MF, of the clinical benefit (defined as prolongation of RBC transfusion intervals by ≥ 50% compared to baseline in transfusion dependent patients or ≥ 1 g/dL Hb increase in the absence of RBC transfusion dependency and/or improvement of at least one MF-associated symptom according by at least 50% and/or improvement of ≥ 2 MF-associated symptoms by at least 25%), progression-free survival, response duration, disease burden (allelic ratio of the respective driver mutation and of high-risk mutations by next-generation sequencing [NGS]), fibrosis grade and overall survival, the quality of life / symptom burden by the Myeloproliferative Neoplasm Symptom Assessment Form (MPN-SAF, MPN10) and the adherence using a modified Morisky 8-item questionnaire.

### Study population

The inclusion and exclusion criteria for this study are outlined in Table 1. Major eligibility criteria include adult female and male patients ≥ 18 years of age diagnosed with myelofibrosis (MF) according to the WHO 2008 or 2016 criteria, including primary (pre-fibrotic or overt) and secondary myelofibrosis. The study focuses on patients with an indication for therapy (either symptomatic patients with splenomegaly > 11 cm diameter and/or symptoms restricting their daily activity or patients with DIPSS int-2, or high risk or MIPSS70 int or high). Patients with failure [secondary resistance] to JAK-inhibitor treatment as defined by IWG-MRT criteria can be enrolled as well as patients with no response or suboptimal response to any JAK-inhibitor therapy (regarding persistence of symptoms, splenomegaly, cytopenia or hyperproliferation) defined either by persisting splenomegaly (> 11 cm total diameter), persisting leukoerythroblastosis, anemia < 6.2 mmol/l (< 10 g/dl), elevated WBC (> 11 Gpt/l) or persisting general or constitutional symptoms (persistence is defined as less than 50% reduction to baseline when using the MPN10 TSS Score).

**Table 1:** Inclusion and Exclusion Criteria

Inclusion Criteria	Patients must meet **ALL** of the following inclusion criteria to be eligible for enrollment into the study: a. Signed Informed Consent Form available b. Patients* ≥ 18 years of age c. Patients diagnosed with myelofibrosis (MF) according to the WHO 2008 or 2016 criteria, including primary (pre-fibrotic or overt) and secondary myelofibrosis d. Patients with an indication for therapy (either symptomatic patients with splenomegaly > 11 cm diameter and/or symptoms restricting their daily activity or patients with DIPSS int-2, or high risk or MIPSS70 int or high) e. Patients with no response or suboptimal response to any JAK-inhibitor therapy (regarding persistence of symptoms, splenomegaly, cytopenia or hyperproliferation) defined either **by ANY ONE of the following criteria** (f-k): f. Persisting Splenomegaly > 11 cm total diameter g. Persisting leukoerythroblastosis h. Anemia < 6.2 mmol/l (< 10 g/dl) i. Elevated WBC (> 11 Gpt/l) j. Persisting general or constitutional symptoms (persistence is defined as less than 50% reduction to baseline when using the MPN10 TSS Score) **OR** k. failure [secondary resistance] to JAK-inhibitor treatment as defined by IWG-MRT criteria l. ECOG performance status < 3 at screening and adequate organ function m. Reliable contraception should be maintained throughout the study and for 1 month after discontinuation of Fedratinib or 5 months after discontinuation of Nivolumab** n. Subject must be willing to receive transfusion of blood products o. Thiamine levels not below lower limit of normal (prior substitution is possible) p. Normal nutritional status, as judged by the physician q. Females of childbearing potential (FCBP) must undergo repetitive pregnancy testing (serum or urine) and pregnancy results must be negative r. Unless practicing complete abstinence from heterosexual intercourse, sexually active FCBP must agree to use adequate contraceptive methods (i.e. failure rate of < 1% per year) s. Males (including those who have had a vasectomy) must use barrier contraception (condoms) when engaging in sexual activity with FCBP. Males must agree not to donate semen or sperm *There are no data that indicate special gender distribution and the risk to be diagnosed with myelofibrosis (MF) does not depend on a patient’s gender. Therefore, patients will be enrolled in the study gender-independently
Exclusion Criteria	The presence of **ANY** of the following criteria will exclude a patient from study enrollment: a. Planned hematopoietic stem cell transplantation within 3 months and suitable donor available b. > 10% blasts in bone marrow smear (cytology) or > 2 × in blood smear within the screening phase or > 20% blasts at any time in bone marrow or peripheral blood smears c. Creatinine > 2xN and Creatinine-Clearance < 45 ml/min; ALAT, ASAT & bilirubin > 3xN (if MF impact on liver > 5xN) d. Baseline platelets count below 50 × 10^9^/L and ANC < 1.0 × 10^9^/L e. Diagnosis of PV, ET (according to WHO 2016) or positive molecular test for BCR-ABL f. Patients on ongoing medication for myelofibrosis including systemic corticosteroids (detailed list of permitted medications is provided in paragraph 9.1.10.4 and Appendix V). Use of steroids within 14 days prior to the first dose of study drug and until end of treatment is prohibited by patients g. Uncontrolled infection h. Evidence of acute or chronic infection with hepatitis B, hepatitis C, human immunodeficiency virus (HIV) or tuberculosis i. Current participation in any other interventional clinical study within 30 days before the first administration of the investigational product or at any time during the study, unless it is an observational (non-interventional) study, or during the follow-up period of an interventional study with last dose of investigational product ≥ 30 days prior first administration of investigational product within this study j. No consent for registration, storage and processing of the individual disease characteristics and course as well as information of the family physician about study participation k. No consent for biobanking of patient’s biological specimens l. Prior therapy with checkpoint-inhibitors m. Vaccination within 4 weeks prior to treatment start n. Hypersensitivity to the IMPs or to any of the excipients o. History of or uncontrolled autoimmune disease such as autoimmune-hepatitis, -pneumonitis, -thyroiditis, chronic inflammatory bowel disease, multiple sclerosis, or rheumatologic diseases (including but not limited to systemic lupus and vasculitis) p. History of malignancy except for i) adequately treated local basal cell or squamous cell carcinoma of the skin, ii) asymptomatic prostate cancer without known metastatic disease and with no requirement for therapy or requiring only hormonal therapy and with normal prostate-specific antigen for ≥ 1 year prior to randomization, or iii) any other cancer that has been in complete remission for ≥ 5 years q. Secondary malignancy that limits survival to less than 6 months r. Drug or alcohol abuse within the last 6 months s. Patients who cannot adhere to the Pregnancy Prevention Plan t. Pregnant or breast-feeding females u. Thiamine levels below normal limit despite supplementation v. Patients who are unable to consent because they do not understand the nature, significance and implications of the clinical trial and therefore cannot form a rational intention in the light of the facts [§ 40 Abs. 1 S. 3 Nr. 3a AMG]

### Study drugs

Fedratinib capsules will be administered orally, QD, 400 mg (100 mg capsules will be provided: 4 capsules of 100 mg per day). Fedratinib treatment will be administered as a run-in phase for 4 weeks. Fedratinib dose modifications will be allowed based on observed toxicity to a 300 mg or 200 mg, or 100 mg daily dose or may be temporarily discontinued in this study. For the first 2 cycles, a fixed dose of 400 mg should be maintained with appropriate supportive medication, as major toxicities have been shown to decrease with increasing number of treatment cycles [31]. Provisions are in place to allow further dose reduction for subjects with co-medication with moderate or strong Cytochrome P4503A4 (CYP3A4) inhibitors.

From cycle 2 on, Nivolumab will be added at a fixed dose of 240 mg i.v., every 2 weeks. The frequency of Nivolumab infusion can be reduced based on observed toxicity. Infusions can be continued even if Fedratinib is temporarily discontinued.

### Study assessments

Treatment response will be evaluated continuously after each treatment cycle (1 cycle = 28 days) according to the IWG-MRT criteria expanded by the response criterion of red cell transfusion (RCT)-independency. In case of progressive disease, study treatment will be stopped; in patients showing response or stable disease with or without clinical benefit (defined as prolongation of RBC transfusion intervals by ≥ 50% compared to baseline in transfusion dependent patients or ≥ 1 g/dL Hb increase in the absence of RBC transfusion dependency and/or improvement of at least one MF-associated symptom according by a minimum of 50% and/or improvement of ≥ 2 MF-associated symptoms by a minimum of 25%) treatment continuation is intended until disease progression or other reasons for withdrawal. Conditions leading to patient withdrawal from the study are detailed in Supplementary Fig. 1. All patients should be screened for inclusion and exclusion criteria within 28 days prior to the first dose of Fedratinib (screening-phase). Detailed study procedures can be found in Table 2.

**Table 2:** Simplified version of schedule of assessments

Protocol activities	Screening/Baseline	On Treatment (visits every 14 days)	EOT	Follow-Up Period (every 3 months until EOS)
Informed consent	X			
Patient data including medical history	X			
Signs/symptoms (PROs)	X	X	X	X
Physical examination	X	X	X	X
PRO	X	X	X	X
Laboratory assessment	X	X	X	X
Thiamine assessment	X	X	X	
Bone marrow biopsy	X	X (cycle 13)	X	X (once per year)

### Outcomes

The primary efficacy endpoint of the study will assess the best response rate within 12 treatment cycles according to the IWG-MRT criteria (including complete remission, CR, partial remission, PR, clinical improvement CI, stable disease, SD) [28], and RCT independency according to Gale et al. [29, 30].

Secondary endpoints include the overall safety profile of Fedratinib and Nivolumab combination characterized by the type, frequency, severity (graded according to the National Cancer Institute Common Terminology Criteria for Adverse Events [NCI CTCAE]), timing and relatedness of adverse events (AEs) and laboratory abnormalities observed during treatment, as well as the cumulative incidence of leukemic transformation, clinical benefit, progression-free survival (PFS), duration of response, overall survival (OS) and reduction of disease burden (Table 3).

**Table 3:** Endpoints

Primary Endpoint Best response rate within 12 treatment cycles according to the IWG-MRT criteria (including complete remission, CR, partial remission, PR, clinical improvement, CI, stable disease, SD [28], and red cell transfusion (RCT) independency according to Gale et al.[29, 30])
Secondary Endpoints • Overall safety profile of Fedratinib and Nivolumab combination characterized by type, frequency, severity (graded using the National Cancer Institute Common Terminology Criteria for Adverse Events [NCI CTCAE]), timing and relatedness of adverse events (AEs) and laboratory abnormalities observed during treatment, as well as cumulative incidence of leukemic transformation • clinical benefit • progression-free survival, • duration of response, • overall survival • reduction of disease burden
Exploratory Endpoints • Quality of life assessed by the Myeloproliferative Neoplasm Symptom Assessment Form (MPN-SAF, MPN10), change in ECOG performance status from study entry to each visit where the variable is measured • Adherence assessed by modified Morisky 8-item questionnaire • Investigation of immune-cell expansion and immune-cell responses to checkpoint-inhibitor therapy at baseline (before first IMP dosing), after 6 months study treatment, and after 12 months study treatment (or at EOT) • Assessment of disease burden measured as allelic burden of the respective driver mutations (JAK2, CALR, MPL) at baseline (before first IMP dosing), after 6 months study treatment and after 12 months study treatment (or at EOT) • Assessment of clonal diversity and evolution by NGS-sequencing of a defined 32 gene panel at baseline (before first IMP dosing), after 6 months study treatment and after 12 months study treatment (or at EOT) • Assessment of bone marrow fibrosis by central histology at baseline before first IMP dosing (screening period) and after 12 months study treatment (or at EOT)

Exploratory endpoints include: Change in ECOG performance status from study entry to each visit at which the variable is measured, investigation of immune-cell expansion and immune-cell responses to checkpoint-inhibitor therapy at baseline (before first IMP dosing), after 6 months study treatment, and after 12 months study treatment (or at end of treatment (EOT)), assessment of disease burden measured as allelic burden of the respective driver mutations (JAK2, CALR, MPL) at baseline (before first investigational medical product (IMP) dosing), after 6 months study treatment and after 12 months study treatment (or at EOT), assessment of clonal diversity and evolution by NGS-sequencing of a defined myeloid gene panel at baseline (before first IMP dosing), after 6 months study treatment and after 12 months study treatment (or at EOT) as well as assessment of bone marrow fibrosis by central histology at baseline before first IMP dosing (screening period) and after 12 months study treatment (or at EOT). Genomic studies will be centrally performed for all patients at study entry. The standard work-up within this trial comprises: i) histomorphology on bone marrow sections including grading of fibrosis and PD-L1 status, ii) gene mutation analyses of *JAK2, MPL*, and *CALR* by Polymerase-chain reaction (PCR), iii) additional genetic analyses such as cytogenetics and the assessment of further gene mutations (e.g. ´High Molecular Risk Marker´ (HMR): *ASXL1, EZH2, SRSF2, IDH1/2*), iv) gene expression analyses of potential biomarkers.

Safety endpoints include hematologic (thrombocytopenia, anemia, and neutropenia) and non-hematological toxicities. All adverse events / toxicities, including serious adverse events, are graded according to NCI CTCAE Version 5.0. Safety assessments will consist of evaluating adverse events, laboratory parameters including hematology and chemistry, vital signs, and physical examinations. Safety will be ensured by internal and external supervision.

### Statistical analyses and sample size

The sample size calculation is based on the A’Hern’s single-stage design [32]. The assumptions used for statistical power calculations are that the true target response rate of 20% (alternative hypothesis) will be tested against the null hypothesis response rate of 5%, with a type I error rate of 0.05 and a power of 80%. A response is defined as achieving a response according to the IWG-MRT criteria. If the number of patients with a response is 4 or more, the null hypothesis is rejected with an actual error rate of alpha < 0.05. If the number of responses is 3 or less, the alternative hypothesis (*P* ≥ 0.2) is rejected with an actual error rate of beta = 0.182 A total of 27 subjects will be needed based on this calculation. A total of 30 patients will need to be recruited in this phase 2 trial assuming an early dropout rate of 10% (*n* = 3). A formal interim analysis will not be performed. The study is conducted at 9 academic centers in Germany (Supplementary Table S1).

## Discussion

Myelofibrosis is a malignant stem-cell disease which is associated with a poor outcome. Overall survival ranges from 5 to 150 months depending on risk factors present at diagnosis [33]. Allogeneic stem cell transplantation remains the only potentially curative treatment option. However, treatment-associated complications are frequent and many patients are not eligible for a transplantation regimen due to age and poor performance status. For these patients, hydroxyurea, androgens, erythropoietin, splenectomy, or splenic radiation are palliative therapeutic options [34]. None of these therapies have been proven to prolong survival in MF, and cytopenia in particular remains an unsolved problem.

The discovery of the Janus kinase (JAK) pathway's role in MF pathogenesis has led to the development of targeted therapies, notably JAK inhibitors. Currently, approval and clinical application of 4 JAK inhibitors (Ruxolitinib, Fedratinib, Momelotinib, and Pacritinib) is established in the treatment of MF. Ruxolitinib, the first JAK inhibitor approved for MF, targets JAK1 and JAK2 pathways. It has been shown to significantly reduce splenomegaly and alleviate symptoms in MF patients, improving overall survival compared to conventional therapies. However, its use is limited by cytopenias, particularly thrombocytopenia and anemia. Momelotinib targets JAK1, JAK2, and ACVR1, thereby addressing both splenomegaly and anemia, a common complication in MF. It stands out for its dual efficacy in improving blood counts while controlling disease symptoms, presenting a valuable option for patients with anemia. Pacritinib is unique among JAK inhibitors for its minimal myelosuppression. It is specifically indicated for patients with severe thrombocytopenia. Pacritinib targets JAK1, JAK2, FLT3, and IRAK1, and has shown efficacy in reducing spleen size and improving symptoms without exacerbating cytopenias.

Fedratinib, a selective JAK2 inhibitor, has been approved for patients with intermediate-2 or high-risk MF, including those previously treated with Ruxolitinib. It offers a beneficial effect on spleen volume and symptom relief. Fedratinib is distinct for its ability to manage patients with a baseline thrombocytopenia, but it requires monitoring of thiamine levels due to the risk of Wernicke's encephalopathy. Fedratinib is an oral small molecule kinase inhibitor with activity against wild type and mutationally activated JAK2 and FLT3 was granted approval for the treatment of myelofibrosis. Fedratinib is a JAK2-selective inhibitor with higher inhibitory activity for JAK2 over family members JAK1, JAK3, and tyrosine kinase 2 (TYK2). Fedratinib demonstrated potent kinase inhibitory activity against JAK2, JAK2V617F and FLT3, with IC50 values of 3 nM, 3 nM and 15 nM, respectively. In contrast, the selectivity for the closely related JAK1, TYK2 and JAK3 is 35-, 135- and > 300-fold lower, respectively, than for JAK2. In contrast to the severe impairment of T-cell function mediated by Ruxolitinib, which has been (in part) attributed to its inhibitory activity on JAK1 [14] and has facilitated its use in diseases with T-cell hyperreactivity such as Graft-versus-Host Disease (GvHD) [35, 36], Fedratinib, does not impair T- and NK-cell responses to a comparable extent [37]. This unique profile of Fedratinib in regard to immune responses facilitates a superior profile regarding immunocompetence of MPN patients in clinical use and the option to combine an effective JAK-inhibitor with immunotherapy.

Besides different JAK-inhibitors with different specificity for JAK1 and JAK2, epigenetic modulators, inhibitors of cell signaling, anti-inflammatory compounds and immunotherapies are within focus of interest in MF. Pre-clinical assessment of checkpoint-inhibitors has shown promising results [20].

Recent phase 2 trials have investigated the use of immune checkpoint-inhibitors in the treatment of myelofibrosis [38, 39] (Table 4). Nivolumab as a single-agent therapy has been investigated in a total of 8 patients. While no severe immune-reactions or adverse events were observed, there was also lack of efficacy beyond stable disease. Notably, only 1/8 patients had a prolonged treatment period of more than 6 months, all other patients had discontinued early due to lack of a meaningful response [38]. In a second trial, Pembrolizumab was investigated as a single-agent therapy in Ruxolitinib pre-treated patients with myelofibrosis [39]. The study followed a Simon 2-stage design and enrolled a total of 10 patients. Pembrolizumab treatment was well tolerated, however, no relevant clinical responses could be recorded, resulting in discontinuation of the trial after the first stage was completed. Immune profiling by flow cytometry, T-cell receptor sequencing, and plasma proteomics demonstrated changes in the immune milieu of patients, which suggested improved T-cell responses that can potentially favor antitumor immunity [39]. At 6 months, only 5/9 chronic phase MF patients were still on treatment.

**Table 4:** Trials investigating checkpoint-inhibitor treatment in MPN

Publication	Phase & Type of Study	Patients	Best Response	Patients on trial at 6 mo	Immune-related AE Grade 3/4
Abou-Dalle I et al. Ann Hem. 2021	Phase II Single-Agent Nivolumab	*n* = 8	Stable Disease	1/8 (12.5%)	None
Hobbs G et al. Blood Adv. 2021	Phase II Single-Agent Pembrolizumab	*n* = 10 (9 CP, 1BP)	Stable Disease	5/9 CP (56%)	None

The fact that patients had no relevant responses regarding spleen size and symptom burden may have contributed to their early discontinuation and drop out (Supplementary Table S2). The combination of checkpoint-inhibitors with the first available JAK-inhibitor Ruxolitinib is not possible due to its efficacy on JAK1 leading to impairment of T-cell function. However, Fedratinib, a JAK2-specific compound, has shown to allow immune responses in pre-clinical and clinical settings. This enables a therapeutic window to explore the induction of immune responses by the established checkpoint-inhibitor Nivolumab in combination with the selective JAK2-inhibitor Fedratinib. Checkpoint-inhibitors mediate indirect anti-tumor activity by activating T-cell responses. While Nivolumab can eradicate malignant MPN cells through activation of T-cell responses, Fedratinib predominantly reduces the spleen size and improves constitutional symptoms. As both aspects of disease control are highly relevant and addressing clinical needs in MF, the combined use of the drugs provides a promising therapeutic concept. Therefore, the risk–benefit ratio is based on arguments discussed above and clearly favors to the beneficial side.

The sequential administration of both drugs is poised to mitigate adverse effects: the initiation of Fedratinib dosing at baseline affords patients the opportunity to acclimate to the treatment and mitigate potential gastrointestinal (GI) toxicity. Notably, GI toxicity is the most common adverse event observed with Fedratinib treatment, as elucidated by England and colleagues [40]. However, its incidence can be significantly attenuated with concurrent interventions such as anti-emetic prophylaxis or standby anti-diarrhea medication like loperamide. Remarkably, while diarrhea manifested in up to 80% of patients during the early Fedratinib trials, the implementation of supportive medication regimens in the FREEDOM trials substantially reduced these side effects to below 40% [40].

Significant discourse revolves around the definition of meaningful endpoints in myelofibrosis trials. The primary endpoint of the FRACTION trial will be the optimal response rate within 12 treatment cycles based on the International Working Group (IWG) criteria, distinguishing it from the endpoints adopted in trials evaluating JAK inhibitors for approval. Historically, studies on JAK inhibitors predominantly focused on reducing spleen size and alleviating symptom burden as primary endpoints, with each subsequent competitor undergoing evaluation within the same framework. Nonetheless, the prognostic impact of spleen size reduction and symptom alleviation remains ambiguous. Although the COMFORT studies [16, 41] demonstrated a survival benefit for Ruxolitinib, despite not being originally powered for this outcome, the definitive disease-modifying potential of these medications remains contentious. Given that allogeneic stem cell transplantation stands as the sole curative option for myelofibrosis, combination therapies should strive for deeper and potentially more clinically significant responses.

### Trial status

Protocol Version 4.0 dated 23 Feb 2023. The first subject was enrolled on July 4, 2022. As of May 2024, 23/30 patients have entered the trial and recruitment is planned to be finished with Last-Patient-First-Visit (LPFV) by December 2024.

### Supplementary Information

Supplementary Material 1: Table S1: List of participating centers. Table S2: Reasons for patient´s withdrawal of treatment.

## Data Availability

Not applicable.

## References

[1] Perner F, Perner C, Ernst T, Heidel FH (2019) Roles of JAK2 in aging, inflammation, hematopoiesis and malignant transformation. Cells 8(8):E854. 10.3390/cells8080854 10.3390/cells8080854 PMC6721738 31398915

[2] Palandri F, Palumbo GA, Bonifacio M, Breccia M, Latagliata R, Martino B, Polverelli N, Abruzzese E, Tiribelli M, Nicolosi M, Bergamaschi M, Tieghi A, Iurlo A, Sgherza N, Cavazzini F, Isidori A, Binotto G, Ibatici A, Crugnola M, Heidel F, Bosi C, Bartoletti D, Auteri G, Catani L, Cuneo A, Aversa F, Semenzato G, Cavo M, Vianelli N, Benevolo G (2018) Durability of spleen response affects the outcome of ruxolitinib-treated patients with myelofibrosis: results from a multicentre study on 284 patients. Leuk Res 74:86–88. 10.1016/j.leukres.2018.10.001 30321784 10.1016/j.leukres.2018.10.001

[3] Palandri F, Palumbo GA, Bonifacio M, Elli EM, Tiribelli M, Auteri G, Trawinska MM, Polverelli N, Benevolo G, Tieghi A, Cavalca F, Caocci G, Beggiato E, Binotto G, Cavazzini F, Miglino M, Bosi C, Crugnola M, Bocchia M, Martino B, Pugliese N, Venturi M, Isidori A, Cattaneo D, Krampera M, Pane F, Cilloni D, Semenzato G, Lemoli RM, Cuneo A, Abruzzese E, Branzanti F, Vianelli N, Cavo M, Heidel F, Iurlo A, Breccia M (2023) A prognostic model to predict ruxolitinib discontinuation and death in patients with myelofibrosis. Cancers (Basel). 15(20):5027. 10.3390/cancers15205027 10.3390/cancers15205027 PMC10605705 37894394

[4] Jayavelu AK, Schnoder TM, Perner F, Herzog C, Meiler A, Krishnamoorthy G, Huber N, Mohr J, Edelmann-Stephan B, Austin R, Brandt S, Palandri F, Schroder N, Isermann B, Edlich F, Sinha AU, Ungelenk M, Hubner CA, Zeiser R, Rahmig S, Waskow C, Coldham I, Ernst T, Hochhaus A, Jilg S, Jost PJ, Mullally A, Bullinger L, Mertens PR, Lane SW, Mann M, Heidel FH (2020) Splicing factor YBX1 mediates persistence of JAK2-mutated neoplasms. Nature 588(7836):157–163. 10.1038/s41586-020-2968-3 33239784 10.1038/s41586-020-2968-3

[5] Lussana F, Cattaneo M, Rambaldi A, Squizzato A (2018) Ruxolitinib-associated infections: a systematic review and meta-analysis. Am J Hematol 93(3):339–347. 10.1002/ajh.24976 29150886 10.1002/ajh.24976

[6] Vannucchi AM, Kiladjian JJ, Griesshammer M, Masszi T, Durrant S, Passamonti F, Harrison CN, Pane F, Zachee P, Mesa R, He S, Jones MM, Garrett W, Li J, Pirron U, Habr D, Verstovsek S (2015) Ruxolitinib versus standard therapy for the treatment of polycythemia vera. N Engl J Med 372(5):426–435. 10.1056/NEJMoa1409002 25629741 10.1056/NEJMoa1409002 PMC4358820

[7] Keohane C, Kordasti S, Seidl T, Perez Abellan P, Thomas NS, Harrison CN, McLornan DP, Mufti GJ (2015) JAK inhibition induces silencing of T Helper cytokine secretion and a profound reduction in T regulatory cells. Br J Haematol 171(1):60–73. 10.1111/bjh.13519 26075866 10.1111/bjh.13519

[8] Parampalli Yajnanarayana S, Stubig T, Cornez I, Alchalby H, Schonberg K, Rudolph J, Triviai I, Wolschke C, Heine A, Brossart P, Kroger N, Wolf D (2015) JAK1/2 inhibition impairs T cell function in vitro and in patients with myeloproliferative neoplasms. Br J Haematol 169(6):824–833. 10.1111/bjh.13373 25824483 10.1111/bjh.13373

[9] Massa M, Rosti V, Campanelli R, Fois G, Barosi G (2014) Rapid and long-lasting decrease of T-regulatory cells in patients with myelofibrosis treated with ruxolitinib. Leukemia 28(2):449–451. 10.1038/leu.2013.296 24145312 10.1038/leu.2013.296

[10] Xing L, Dai Z, Jabbari A, Cerise JE, Higgins CA, Gong W, de Jong A, Harel S, DeStefano GM, Rothman L, Singh P, Petukhova L, Mackay-Wiggan J, Christiano AM, Clynes R (2014) Alopecia areata is driven by cytotoxic T lymphocytes and is reversed by JAK inhibition. Nat Med 20(9):1043–1049. 10.1038/nm.3645 25129481 10.1038/nm.3645 PMC4362521

[11] Wang SP, Iwata S, Nakayamada S, Sakata K, Yamaoka K, Tanaka Y (2014) Tofacitinib, a JAK inhibitor, inhibits human B cell activation in vitro. Ann Rheum Dis 73(12):2213–2215. 10.1136/annrheumdis-2014-205615 25157177 10.1136/annrheumdis-2014-205615

[12] Rizzi M, Lorenzetti R, Fischer K, Staniek J, Janowska I, Troilo A, Strohmeier V, Erlacher M, Kunze M, Bannert B, Kyburz D, Voll RE, Venhoff N, Thiel J (2017) Impact of tofacitinib treatment on human B-cells in vitro and in vivo. J Autoimmun 77:55–66. 10.1016/j.jaut.2016.10.005 27793425 10.1016/j.jaut.2016.10.005

[13] Heine A, Held SA, Daecke SN, Wallner S, Yajnanarayana SP, Kurts C, Wolf D, Brossart P (2013) The JAK-inhibitor ruxolitinib impairs dendritic cell function in vitro and in vivo. Blood 122(7):1192–1202. 10.1182/blood-2013-03-484642 23770777 10.1182/blood-2013-03-484642

[14] Perner F, Schnoder TM, Ranjan S, Wolleschak D, Ebert C, Pils MC, Frey S, Polanetzki A, Fahldieck C, Schonborn U, Schraven B, Isermann B, Fischer T, Heidel FH (2016) Specificity of JAK-kinase inhibition determines impact on human and murine T-cell function. Leukemia 30(4):991–995. 10.1038/leu.2015.218 26242463 10.1038/leu.2015.218

[15] Verstovsek S, Mesa RA, Gotlib J, Gupta V, DiPersio JF, Catalano JV, Deininger MW, Miller CB, Silver RT, Talpaz M, Winton EF, Harvey JH Jr, Arcasoy MO, Hexner EO, Lyons RM, Paquette R, Raza A, Jones M, Kornacki D, Sun K, Kantarjian H, C.-I. investigators, (2017) Long-term treatment with ruxolitinib for patients with myelofibrosis: 5-year update from the randomized, double-blind, placebo-controlled, phase 3 COMFORT-I trial. J Hematol Oncol 10(1):55. 10.1186/s13045-017-0417-z 28228106 10.1186/s13045-017-0417-z PMC5322633

[16] Verstovsek S, Mesa RA, Gotlib J, Levy RS, Gupta V, DiPersio JF, Catalano JV, Deininger M, Miller C, Silver RT, Talpaz M, Winton EF, Harvey JH Jr, Arcasoy MO, Hexner E, Lyons RM, Paquette R, Raza A, Vaddi K, Erickson-Viitanen S, Koumenis IL, Sun W, Sandor V, Kantarjian HM (2012) A double-blind, placebo-controlled trial of ruxolitinib for myelofibrosis. N Engl J Med 366(9):799–807. 10.1056/NEJMoa1110557 22375971 10.1056/NEJMoa1110557 PMC4822164

[17] Verstovsek S, Mesa RA, Gotlib J, Levy RS, Gupta V, DiPersio JF, Catalano JV, Deininger MW, Miller CB, Silver RT, Talpaz M, Winton EF, Harvey JH Jr, Arcasoy MO, Hexner EO, Lyons RM, Paquette R, Raza A, Vaddi K, Erickson-Viitanen S, Sun W, Sandor V, Kantarjian HM (2013) Efficacy, safety and survival with ruxolitinib in patients with myelofibrosis: results of a median 2-year follow-up of COMFORT-I. Haematologica 98(12):1865–1871. 10.3324/haematol.2013.092155 24038026 10.3324/haematol.2013.092155 PMC3856961

[18] Harrison CN, Schaap N, Vannucchi AM, Kiladjian JJ, Tiu RV, Zachee P, Jourdan E, Winton E, Silver RT, Schouten HC, Passamonti F, Zweegman S, Talpaz M, Lager J, Shun Z, Mesa RA (2017) Janus kinase-2 inhibitor fedratinib in patients with myelofibrosis previously treated with ruxolitinib (JAKARTA-2): a single-arm, open-label, non-randomised, phase 2, multicentre study. Lancet Haematol 4(7):e317–e324. 10.1016/S2352-3026(17)30088-1 28602585 10.1016/S2352-3026(17)30088-1 PMC8207822

[19] Palandri F, Latagliata R, Polverelli N, Tieghi A, Crugnola M, Martino B, Perricone M, Breccia M, Ottaviani E, Testoni N, Merli F, Aversa F, Alimena G, Cavo M, Martinelli G, Catani L, Baccarani M, Vianelli N (2015) Mutations and long-term outcome of 217 young patients with essential thrombocythemia or early primary myelofibrosis. Leukemia 29(6):1344–1349. 10.1038/leu.2015.87 25801912 10.1038/leu.2015.87

[20] Prestipino A, Emhardt AJ, Aumann K, O'Sullivan D, Gorantla SP, Duquesne S, Melchinger W, Braun L, Vuckovic S, Boerries M, Busch H, Halbach S, Pennisi S, Poggio T, Apostolova P, Veratti P, Hettich M, Niedermann G, Bartholoma M, Shoumariyeh K, Jutzi JS, Wehrle J, Dierks C, Becker H, Schmitt-Graeff A, Follo M, Pfeifer D, Rohr J, Fuchs S, Ehl S, Hartl FA, Minguet S, Miething C, Heidel FH, Kroger N, Triviai I, Brummer T, Finke J, Illert AL, Ruggiero E, Bonini C, Duyster J, Pahl HL, Lane SW, Hill GR, Blazar BR, von Bubnoff N, Pearce EL, Zeiser R (2018) Oncogenic JAK2(V617F) causes PD-L1 expression, mediating immune escape in myeloproliferative neoplasms. Sci Transl Med 10(429). 10.1126/scitranslmed.aam7729

[21] Medication Guide INREBIC®(fedratinib) (2019) [cited 2021 September]; Available from: https://www.accessdata.fda.gov/drugsatfda_docs/label/2019/212327s000lbl.pdf. Accessed 08.02.2021

[22] Investigator's Brochure Nivolumab (2020) [cited 2021 September]; Available from: https://www.ctc.ucl.ac.uk/TrialDocuments/Uploaded/BMS%20Nivolumab%20IB%20v19_tracked%20changes_17112020_0.pdf. Accessed 19 Jun 2015

[23] Animesh Pardanani RS, Talpaz M, Cortes J, Gotlib J, Jamieson C, Rose S, Berry T, Zhang J, Tefferi A (2020) Long-term safety of fedratinib in patients with intermediate- or high-risk myelofibrosis (MF), in 62nd ASH annual meeting

[24] Harrison CN, Schaap N, Vannucchi AM, Kiladjian JJ, Jourdan E, Silver RT, Schouten HC, Passamonti F, Zweegman S, Talpaz M, Verstovsek S, Rose S, Shen J, Berry T, Brownstein C, Mesa RA (2020) Fedratinib in patients with myelofibrosis previously treated with ruxolitinib: An updated analysis of the JAKARTA2 study using stringent criteria for ruxolitinib failure. Am J Hematol 95(6):594–603. 10.1002/ajh.25777 32129512 10.1002/ajh.25777 PMC7317815

[25] Hotchkiss RS, Colston E, Yende S, Crouser ED, Martin GS, Albertson T, Bartz RR, Brakenridge SC, Delano MJ, Park PK, Donnino MW, Tidswell M, Mayr FB, Angus DC, Coopersmith CM, Moldawer LL, Catlett IM, Girgis IG, Ye J, Grasela DM (2019) Immune checkpoint inhibition in sepsis: a phase 1b randomized study to evaluate the safety, tolerability, pharmacokinetics, and pharmacodynamics of nivolumab. Intensive Care Med 45(10):1360–1371. 10.1007/s00134-019-05704-z 31576433 10.1007/s00134-019-05704-z PMC9006384

[26] Pardanani A, Harrison C, Cortes JE, Cervantes F, Mesa RA, Milligan D, Masszi T, Mishchenko E, Jourdan E, Vannucchi AM, Drummond MW, Jurgutis M, Kuliczkowski K, Gheorghita E, Passamonti F, Neumann F, Patki A, Gao G, Tefferi A (2015) Safety and efficacy of fedratinib in patients with primary or secondary myelofibrosis: a randomized clinical trial. JAMA Oncol 1(5):643–651. 10.1001/jamaoncol.2015.1590 26181658 10.1001/jamaoncol.2015.1590

[27] Watanabe E, Nishida O, Kakihana Y, Odani M, Okamura T, Harada T, Oda S (2020) Pharmacokinetics, pharmacodynamics, and safety of Nivolumab in patients with Sepsis-Induced Immunosuppression: a multicenter, open-label phase 1/2 study. Shock 53(6):686–694. 10.1097/SHK.0000000000001443 31513050 10.1097/SHK.0000000000001443 PMC7448837

[28] Tefferi A, Barosi G, Mesa RA, Cervantes F, Deeg HJ, Reilly JT, Verstovsek S, Dupriez B, Silver RT, Odenike O, Cortes J, Wadleigh M, Solberg LA Jr., Camoriano JK, Gisslinger H, Noel P, Thiele J, Vardiman JW, Hoffman R, Cross NC, Gilliland DG, Kantarjian H, IWGfM Research, and Treatment (2006) International Working Group (IWG) consensus criteria for treatment response in myelofibrosis with myeloid metaplasia, for the IWG for Myelofibrosis Research and Treatment (IWG-MRT). Blood 108(5):1497–503. 10.1182/blood-2006-03-009746 16675707 10.1182/blood-2006-03-009746

[29] Gale RP, Barosi G, Barbui T, Cervantes F, Dohner K, Dupriez B, Gupta V, Harrison C, Hoffman R, Kiladjian JJ, Mesa R, Mc Mullin MF, Passamonti F, Ribrag V, Roboz G, Saglio G, Vannucchi A, Verstovsek S (2011) What are RBC-transfusion-dependence and -independence? Leuk Res 35(1):8–11. 10.1016/j.leukres.2010.07.015 20692036 10.1016/j.leukres.2010.07.015 PMC8215731

[30] Gale RP, Barosi G, Barbui T, Cervantes F, Dohner K, Dupriez B, Gupta V, Harrison C, Hoffman R, Kiladjian JJ, Mesa R, Mc Mullin MF, Passamonti F, Ribrag V, Roboz G, Saglio G, Vannucchi A, Verstovsek S (2012) RBC-transfusion guidelines update. Leuk Res 36(5):659–660. 10.1016/j.leukres.2012.01.023 22336392 10.1016/j.leukres.2012.01.023 PMC8162055

[31] Pardanani A, Stone RM, Talpaz M, Cortes J, Gotlib J, Jamieson C, Rose SA, Berry T, Zhang J, Tefferi (2020) Long-term safety of fedratinib in patients with intermediate- or high-risk myelofibrosis (MF). Hemasphere (EHA Annual Meeting Abstracts 2020). (Abstract release date: 05/14/20) EHA Library. 06/12/2020; 293585; EP1096

[32] A’Hern RP (2001) Sample size tables for exact single-stage phase II designs. Stat Med 20(6):859–866. 10.1002/sim.721 11252008 10.1002/sim.721

[33] Guglielmelli P, Lasho TL, Rotunno G, Mudireddy M, Mannarelli C, Nicolosi M, Pacilli A, Pardanani A, Rumi E, Rosti V, Hanson CA, Mannelli F, Ketterling RP, Gangat N, Rambaldi A, Passamonti F, Barosi G, Barbui T, Cazzola M, Vannucchi AM, Tefferi A (2018) MIPSS70: mutation-enhanced international prognostic score system for transplantation-age patients with primary myelofibrosis. J Clin Oncol 36(4):310–318. 10.1200/JCO.2017.76.4886 29226763 10.1200/JCO.2017.76.4886

[34] Griesshammer M, Baerlocher GM, Döhner K, Gisslinger H, Koschmieder S, Petrides PE, Lengfelder E (2018) Primäre Myelofibrose (PMF). Onkopedia Leitlinien [cited 2021 August]; Available from: https://www.onkopedia.com/de/onkopedia/guidelines/primaere-myelofibrose-pmf/@@guideline/html/index.html. Accessed 31 Aug 2021

[35] Zeiser R, Burchert A, Lengerke C, Verbeek M, Maas-Bauer K, Metzelder SK, Spoerl S, Ditschkowski M, Ecsedi M, Sockel K, Ayuk F, Ajib S, de Fontbrune FS, Na IK, Penter L, Holtick U, Wolf D, Schuler E, Meyer E, Apostolova P, Bertz H, Marks R, Lubbert M, Wasch R, Scheid C, Stolzel F, Ordemann R, Bug G, Kobbe G, Negrin R, Brune M, Spyridonidis A, Schmitt-Graff A, van der Velden W, Huls G, Mielke S, Grigoleit GU, Kuball J, Flynn R, Ihorst G, Du J, Blazar BR, Arnold R, Kroger N, Passweg J, Halter J, Socie G, Beelen D, Peschel C, Neubauer A, Finke J, Duyster J, von Bubnoff N (2015) Ruxolitinib in corticosteroid-refractory graft-versus-host disease after allogeneic stem cell transplantation: a multicenter survey. Leukemia 29(10):2062–2068. 10.1038/leu.2015.212 26228813 10.1038/leu.2015.212 PMC4854652

[36] Zeiser R, von Bubnoff N, Butler J, Mohty M, Niederwieser D, Or R, Szer J, Wagner EM, Zuckerman T, Mahuzier B, Xu J, Wilke C, Gandhi KK, Socie G, Group RT (2020) Ruxolitinib for glucocorticoid-refractory acute graft-versus-host disease. N Engl J Med 382(19):1800–1810. 10.1056/NEJMoa1917635 32320566 10.1056/NEJMoa1917635

[37] Ahsan A, La Motte-Mohs R, Hagner P, Thakurta A (2021) Fedratinib demonstrates limited suppression of t- and Natural Killer- (NK) cell activity ex vivo compared with ruxolitinib at clinically relevant doses. Hemasphere (EHA annual meeting abstracts 2021). EHA Library. Ahsan A. 06/09/21; 324785; EP1062

[38] Abou Dalle I, Kantarjian H, Daver N, Masarova L, Pemmaraju N, Bose P, Garcia-Manero G, Verstovsek S (2021) Phase II study of single-agent nivolumab in patients with myelofibrosis. Ann Hematol 100(12):2957–2960. 10.1007/s00277-021-04618-5 34350483 10.1007/s00277-021-04618-5

[39] Hobbs G, Cimen Bozkus C, Moshier E, Dougherty M, Bar-Natan M, Sandy L, Johnson K, Foster JE, Som T, Macrae M, Marble H, Salama M, El Jamal SM, Zubizarreta N, Wadleigh M, Stone R, Bhardwaj N, Iancu-Rubin C, Mascarenhas J (2021) PD-1 inhibition in advanced myeloproliferative neoplasms. Blood Adv 5(23):5086–5097. 10.1182/bloodadvances.2021005491 34581778 10.1182/bloodadvances.2021005491 PMC9152999

[40] England JT, Gupta V (2022) Fedratinib: a pharmacotherapeutic option for JAK-inhibitor naive and exposed patients with myelofibrosis. Expert Opin Pharmacother 23(15):1677–1686. 10.1080/14656566.2022.2135989 36252265 10.1080/14656566.2022.2135989

[41] Harrison C, Kiladjian JJ, Al-Ali HK, Gisslinger H, Waltzman R, Stalbovskaya V, McQuitty M, Hunter DS, Levy R, Knoops L, Cervantes F, Vannucchi AM, Barbui T, Barosi G (2012) JAK inhibition with ruxolitinib versus best available therapy for myelofibrosis. N Engl J Med 366(9):787–798. 10.1056/NEJMoa1110556 22375970 10.1056/NEJMoa1110556

