# Supplementary material for: FRACTION: protocol of a phase II study of Fedratinib and Nivolumab combination in patients with myelofibrosis and resistance or suboptimal response to JAK-inhibitor treatment of the German MPN study group (GSG-MPN)
Source: Ann Hematol. 2024 Jul 5;103(8):2775–85. doi: 10.1007/s00277-024-05867-w (PMC11283433; doi:10.1007/s00277-024-05867-w)
Supplement: Supplementary file 1 — Supplementary Material 1: Table S1: List of participating centers. Table S2: Reasons for patient´s withdrawal of treatment. [file 277_2024_5867_MOESM1_ESM.docx]

**FRACTION:** **Protocol of a Phase II Study of Fedratinib and Nivolumab Combination in Patients with Myelofibrosis and Resistance or Suboptimal Response to JAK-inhibitor Treatment of the German MPN Study Group (GSG-MPN)**

Susanne Isfort^1^, Nikolas von Bubnoff^2^, Haifa Kathrin Al-Ali^3^, Heiko Becker^4^, Thorsten Götze^5,6^, Philipp le Coutre^7^, Martin Griesshammer^8^, Claudia Moskwa^9^, Luisa Wohn^6^, Johanna Riedel^6^, Francesca Palandri^10^, Kirsi Manz^1,11^, Andreas Hochhaus^12^, Konstanze Döhner^13^ and Florian H. Heidel^1,9^

**Supplementary Data**

**Table S1: List of participating centers**

| Center | PI |
| --- | --- |
| University Hospital Halle/Saale | Prof. Dr. H.K. Al-Ali |
| University Hospital Ulm | Prof. Dr. K. Döhner |
| University Hospital Greifswald | Dr. C. Moskwa |
| Frankfurt Nordwest Hospital | Prof. Dr. Th. Götze |
| University Hospital Lübeck | Prof. Dr. N. von Bubnoff |
| J.-Weseling-Hospital Minden (Bochum University) | Prof. Dr. M. Griesshammer |
| Charite University Hospital, Berlin | Prof. Dr. Ph. le Coutre |
| University Hospital Freiburg | Prof. Dr. H. Becker |
| Hannover Medical School (MHH) | Prof. Dr. F. Heidel |

**Table S2: Reasons for patient´s withdrawal of treatment**

Patients may continue with therapy unless any of the following occurs:

 Progressive disease at any time

 Unacceptable toxicity dictating cessation of treatment (if only one substance needs to be discontinued, administration of the other IMP can be continued).

 Changes in medical status of the patient such that the investigator believes that patient safety will be compromised or that it would be in the best interest of the patient to stop treatment.

 Pregnancy

 Withdrawal of patient’s consent to continue therapy. If the patient withdraws consent to continue therapy, EOT should be documented and the patient should be followed up

 Non-compliance by the patient with protocol requirements.

 Patient is lost to follow-up. If a patient does not return for scheduled visits, every effort should be made to re-establish contact. In any circumstance, every effort should be made to document patient outcome, if possible.

 The drug combination becomes commercially available.

 termination of the whole study by the Sponsor

 Maximum treatment duration of 12 months reached

In summary, a patient should be withdrawn from the trial treatment if, in the opinion of the investigator, it is medically necessary, or if it is the wish of the patient, or the whole study is terminated.

After end of treatment, the patient will be removed from the active study part and will enter the follow-up phase for safety and survival status until death, end of the whole study or patient´s wish.

The Sponsor may request that survival data be collected on all enrolled subjects outside of the protocol defined window. At the time of this request, each subject will be contacted to determine their survival status unless the subject has withdrawn consent for all contact.
